# Outbreak of *cryptosporidium hominis* following river flooding in the city of Halle (Saale), Germany, August 2013

**DOI:** 10.1186/s12879-015-0807-1

**Published:** 2015-02-22

**Authors:** Maximilian Gertler, Matthias Dürr, Peter Renner, Sven Poppert, Mona Askar, Janina Breidenbach, Christina Frank, Karina Preußel, Anika Schielke, Dirk Werber, Rachel Chalmers, Guy Robinson, Irmgard Feuerpfeil, Egbert Tannich, Christine Gröger, Klaus Stark, Hendrik Wilking

**Affiliations:** Department for Infectious Disease Epidemiology, Robert Koch Institute (RKI), Seestraße 10, 13353 Berlin, Germany; Postgraduate Training for Applied Epidemiology, affiliated to the European Programme for Intervention Epidemiology Training, European Centres of Disease Controle (ECDC), Sweden, Europe; Public Health authority Halle (Saale), Niemeyerstraße 1, 06110 Halle (Saale), Germany; Federal Environment Protection Agency (UBA), Heinrich-Heine-Str. 12, 08645 Bad Elster, Germany; National Reference Centre for Tropical Pathogens, Bernhard Nocht Institute for Tropical Medicine (BNI), Bernhard-Nocht-Straße 74, 20359 Hamburg, Germany; Cryptosporidium Reference Unit, Public Health Wales Microbiology, Singleton Hospital, Swansea, SA2 8QA UK; Justus-Liebig-University Giessen, Institute of Medical Microbiology, Giessen, Germany

**Keywords:** Waterborne outbreak, Outbreak investigation, River flooding, Water sampling, Cryptosporidium hominis, Case–control study, Bathing

## Abstract

**Background:**

During weeks 32–33, 2013, 24 cases of cryptosporidiosis were notified in the city of Halle (annual mean 2008–2012: 9 cases). We investigated the outbreak to identify the source and recommend control measures, considering that between weeks 23–25 the river Saale which flows through the city centre overflowed the floodplain, parts of the city centre and damaged sewage systems.

**Methods:**

We defined a case as a resident of Halle with gastroenteritis, *Cryptosporidium*-positive stool and disease onset weeks 27 through 47. In a case–control study among kindergarten children, we compared cases and controls regarding environmental exposure, use of swimming pools, zoo visits and tap water consumption 14 days pre-onset or a corresponding 14-days-period (controls) and adjusted for residence. Stool specimens were tested by microscopy and PCR, and *Cryptosporidium* DNA was sequenced. Samples from public water system, swimming pools and river Saale were examined for *Cryptosporidium* oocysts (microscopy and PCR).

**Results:**

Overall, 167 cases were detected, 40/167 (24%) were classified as secondary cases. First disease onsets occurred during week 29, numbers peaked in week 34 and started to decrease in week 36. Median age was 8 years (range: 0–77). Compared to controls (n = 61), cases (n = 20) were more likely to report visits to previously flooded areas (OR: 4.9; 95%-CI: 1.4-18) and the zoo (OR: 2.6; 95%-CI: 0.9-7.6). In multivariable analysis visits to the floodplain remained the sole risk factor (OR: 5.5; 95%-CI: 1.4-22). Only *C.hominis* of a single genotype (IbA9G2) was detected in stools. Oocysts were detected in samples from the river, two local lakes and three public swimming pools by microscopy, but not in the public water supply.

**Conclusions:**

Evidence suggests that activities in the dried out floodplain led to infection among children. Secondary transmissions may be involved. Consequently, authorities recommended to avoid playing, swimming and having picnics in the flood-affected area. Health authorities should consider the potential health risks of long-term surviving parasites persisting on flooded grounds and in open waters even several weeks after the flooding and of bathing places close to sewage spill-overs. Preventive measures comprise water sampling (involving parasites), information of the public and prolonged closures of potentially contaminated sites.

## Background

The protozoa in the genus *Cryptosporidium* are important parasitic pathogens causing water and food contamination leading to diarrhoeal disease [1]. Waterborne outbreaks occur globally and have been linked to recreational water exposure and drinking water consumption. Contamination of the waters has been shown to be due to human (wastewater) and animal (livestock and wildlife) sources [2,3].

Clinically, cryptosporidiosis may cause profuse and long-lasting (usually 1–2 weeks, ranging from a few days to 4 weeks) but usually self-limiting diarrhoea with abdominal cramping. Chronic or systemic infections are reported in immunodeficient patients. Treatment is usually symptomatic, with no licensed specific therapy in the EU. Repeat infections elicit some degree of immunity [1,4].

To date, around 30 species in the genus are identified.Disease in humans is predominantly caused by *Cryptosporidium* (*C.*) *parvum* and *hominis* [5]. While *C. parvum* infects a broad spectrum of mammals, *C.hominis* has a human infection cycle [2,6]. The oocysts of the parasite represent the infective form which can survive and remain infective for many months outside a host, particularly in moist and cool environments. Oocysts can survive in river sediments and rivers were demonstrated to have acted as transfer medium from livestock to public water supplies. The pathogen is not sensitive to chlorine disinfection as applied in drinking water treatments. Transmission takes place by oral ingestion. The incubation period is 2–10 days and infected persons can excrete the pathogens for several months after symptoms have stopped [7].

In Germany, by federal law all diagnostic laboratories are obliged to notify the local public health departments of patients with stool tests positive for *Cryptosporidium*. Between 900 to 1.400 cases have been reported annually between 2008 and 2012 in Germany. Outbreaks resulting in notification were restricted to households or community settings [8].

## Environmental context

In late May and early June 2013, extreme river flooding in Eastern Germany began after several days of heavy rain. The river Saale, a tributary of the river Elbe and navigable for inland water transport, overflowed the floodplain and parts of the city centre of Halle (inhabitants 230,000), Saxony Anhalt, Germany. The flooding peaked on 5^th^ June and the city council declared the state of disaster from 3^rd^ to 8^th^ June. The flooding was considered the worst since 1890. It affected central parts of the sewage piping system and damaged sewage pumps in several locations along the river and the floodplain. A central sewage collector tunnel running along the river bank normally removes the wastewaters to a treatment plant northwards and downriver of the city centre. Numerous emergency spillways allow direct efflux from the tunnel into the river in case of extremely high water levels e.g. after heavy rainfall or in case of flooding.

The floodplain bisecting the city represents the central recreational area for the urban population of Halle and includes playgrounds, ponds, parks and sports facilities. Bathing, although officially not approved, is very common at a river beach and other locations, and children have easy water access to the normally gently flowing stream. The area was polluted by the floods and consequently closed to the public for cleaning. The area was reopened to the public in stages from mid-July on and the beach use resumed during the last week of July. The summer holidays in Halle started on 15 July 2013. A period with much sunny and hot summer weather lasted from early July until end of August 2013, likely encouraging the use of the recreational areas in the reopened floodplain.

## Outbreak signals

During calendar weeks 32 and 33 (5–18 August), 2013, 24 cases of cryptosporidiosis were notified in the city of Halle. By comparison the annual average in years 2008–12 in Halle was 9 cases. This increase was simultaneously detected by the local health department in Halle and by the Robert Koch Institute (RKI) in Berlin, the federal level public health institute in Germany, through application of electronic routine cluster algorithm for outbreak detection. An outbreak investigation team was formed and included public health professionals from the local health department, expert microbiologists for drinking water and recreational water from the German Federal Environmental Agency (UBA) in Bad Elster, an expert parasitologist from the Bernhard Nocht Institute for Tropical Medicine in Hamburg and epidemiologists from the Robert Koch-Institute (RKI). This report describes the epidemiological and microbiologic investigations which aimed to identify the source of the outbreak and recommend control measures.

## Methods

### Epidemiological investigation

#### Initial assessment

For confirmation of the outbreak, laboratories involved in primary stool diagnostics in Halle as well as local general practitioners and paediatricians in Halle were interviewed regarding current sample loads of incoming diarrhoea stools, routine test procedures, laboratory testing frequency and the number (and perceived trends in numbers) of patients presenting with diarrhoea.

### Descriptive epidemiology and case finding

An outbreak case was defined a case as a resident of Halle with gastroenteritis (diarrhoea or vomiting or abdominal cramps) and *Cryptosporidium*-positive stool (microscopy or antigen detection) with disease onset weeks 27 through 47 and who did not stay outside Halle for more than 7 out of the 2 to 14 days incubation period . Secondary outbreak cases were defined as cases with reported contact to persons with diarrhoea in either their household, kindergarten or school during the two weeks prior to onset of symptoms. The local health authorities published a press release calling on individuals with diarrhoea to see their doctor for stool sampling.

### Hypothesis generation

Cases and parents of infant cases were routinely contacted by the public health authority of Halle regarding symptom onset, contact to other cases of gastroenteritis and general exposure assessment as attendance of kindergarten, schools or at other communal facilities. Additionally, a sample of primary cases was interviewed using a standardised explorative questionnaire concerning: disease history, traveling, consumption of drinking water, visits to specific public pools and lakes, visits of play grounds with fountains, animal exposure, activities in previously flooded area, general eating habits, usual grocery shopping and visits of public food places (e.g. ice cream vendors).

### Case control study

The objective of the case–control study was to test the main hypothesized sources/modes for infection with cryptosporidiosis: activities in the floodplain, visits to swimming pools and such pools where oocysts where detected, the consumption of tap and mineral water and visits to the zoo in the 14 days before symptom-onset.

We recruited primary cases and controls among kindergarten children because this subgroup represented 39/80 (49%) of all primary cases until the start of the study on 5 September. Also they were considered to be approachable comparably quickly via day-care centres. All kindergartens in Halle were informed by the city council and were asked to inform the parents about the study using information material provided by the outbreak investigation team. Cases’ parents were interviewed by employees of the RKI via telephone.

The information material provided to the parents contained a weblink to an online questionnaire which could be filled in autonomously and voluntarily.

Control persons were defined as children without symptoms of gastroenteritis (vomiting or diarrhoea) after 1 July 2013 and who did not have a history of travel outside Halle for 7 days or more during the 14 days before symptom-onset. The questionnaire was accessible between 5 and 16 September 2013. For the questioning of control persons we defined a reference observation period from 29 July to 11 August 2013.

Analysis utilised univariable logistic regression adjusted for district of residence. Candidate variables for multivariable logistic regression had a p-value smaller than 0.1 in the univariable analysis. This was calculated using STATA 12.1 (StataCorp LP). In accordance with article 25, section 1, of the German Infection Protection Act of 2001, a formal ethical review process and approval was not required for this investigation of an ongoing outbreak. Data protection and medical ethics standards were adhered to as regulated by law. Parents of controls received a detailed information letter about the study with the invitation to participate online, parents of cases were asked verbal consent before start of the telephone interview.

### Microbiological investigation

#### Human faecal samples

*Cryptosporidium*-positive faecal samples were referred to the National reference centre for tropical pathogens (NRC) for confirmation of diagnosis and species identification. For confirmation of the genus *Cryptosporidium*, stool microscopy was performed using faecal smears following formol-ether concentration of stool samples [9] and subsequent staining by the modified cold Kinyoun acid fast method [10]. In addition, multiplex PCR was performed able to detect *Cryptosporidium* spp., *Giardia lamblia, Entamoeba histolytica* and *Cyclospora cayetanenesis* DNA in faecal samples [11,12]. For species and subtype identification of *Cryptosporidiae*, sequencing of PCR amplified DNA fragments of the 18S rRNA and the 60 kDa glycoprotein (GP60) genes was performed [13-16].

### Environmental samples

**Figure 1:**
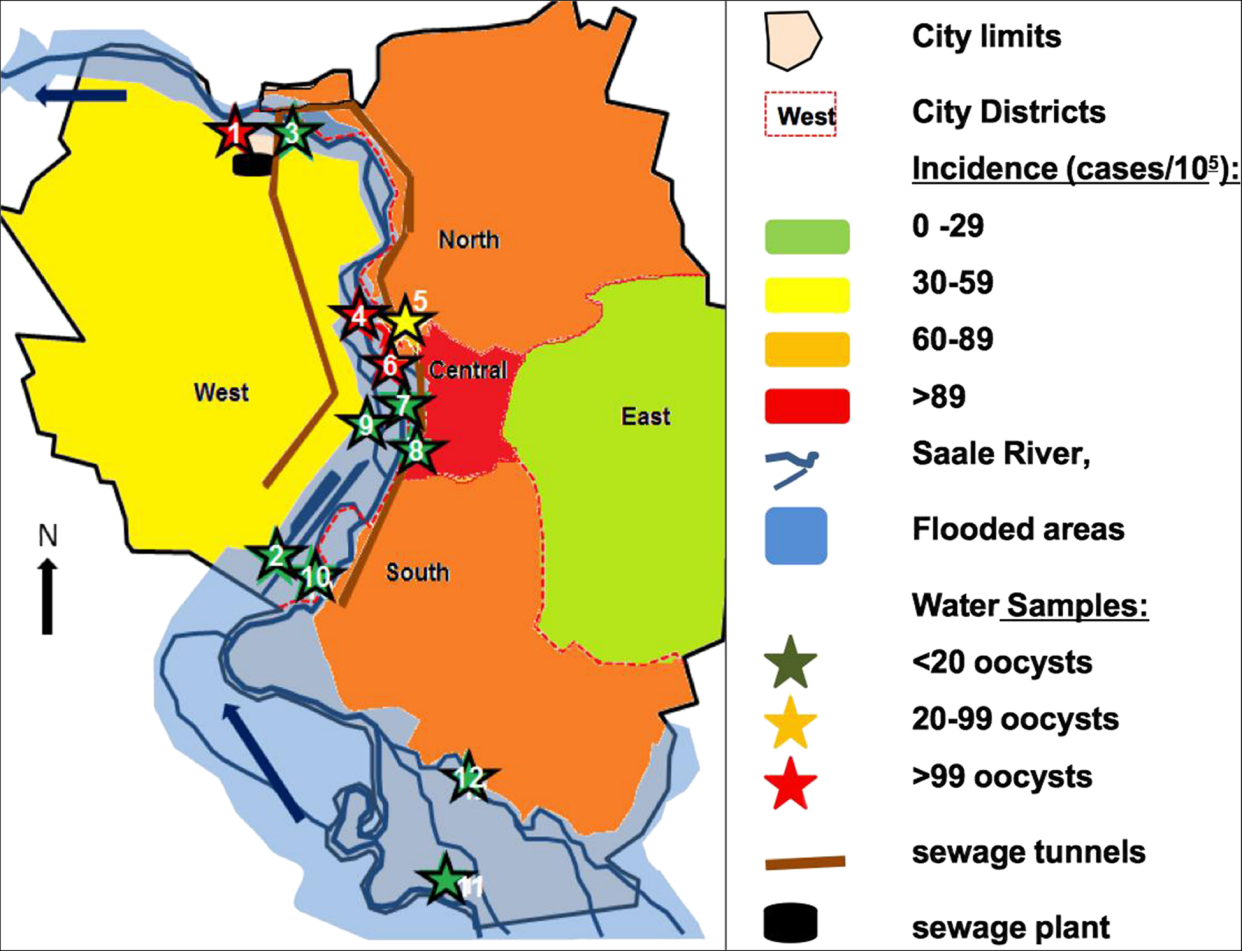
**Schematic map of the city of Halle and the Saale river.**

In order to trace the source and to monitor the faecal pollution 5 samples were taken from the public drinking water system (27 August 2013), 13 samples from pools of public baths, 12 samples from 10 different sites in the river Saale including lateral branches and 5 samples from other bodies of water in Halle (for details see Figure 1 and Table 1).

**Table 1:** **Concentration of** ***Cryptosporidium*** **oocysts (oocysts/100 litres) in open waters in the floodplain and in public baths in Halle, Germany 2013**

**No. on map**	**Sampling spot**	**Bathing water**	**Number of oocysts date of sampling**				
			**27 Aug**	**2 Sept**	**11 Sept**	**24 Sept**	**11 Nov**
1	Pond in floodplain (in Kröllwitz)	n			1		
2	Pond in floodplain (in Regattastrecke)	n			0		
3	Pond in floodplain (Planena)	n			0		
4	River Saale (near sewage plant North)	n				148	
5	Saale lateral channel Mühlgraben (Street Ochsenbrücke, near beach)	y^2^			592	460	4
6	Saale lateral channel Mühlgraben (150 m up-stream from Street Ochsenbrücke)	n				20	
7	Saale lateral channel Mühlgraben (300 m up-stream from Ochsenbrücke, West)	n					3
8	Saale lateral channel Mühlgraben (300 m upstream from Ochsenbrücke East)	n					144
9	Saale lateral channel Mühlgraben (500 m upstream from Ochsenbrücke)	n					5
10	Saale tributary Wilde Saale (in Gimritz)	n				5	
11	River Saale (in island Rabeninsel)	n				0	
12	River Saale (in island Planena	n				4	
	Saale tributary Weiße Elster	n				7	
	Pond outside floodplain	y	1				
	Pond outside floodplain	y		0			
	Public bath N	y	99 / 0^1^				
	Public bath M	y		0/0/3^1^	0/0/1/3^1^		
	Public bath S	y	11 / 0^1^				36 / 100^1^

^1^From different pools.

^2^Bathing officially not approved; y = yes, n = no, m = meter.

Sampling and analysis were carried out according to international standards [17]. A portable sampling device was used to pump water samples of 100 l through a membrane filter (PALL-Envirocheck ®). In the laboratory, the oocysts were eluted from the filters with a buffer-tenside mixture on a shaking apparatus. The eluate was centrifuged and the formed pellet was resuspended in 10 ml of buffer. The oocysts were extracted from the suspension by immunomagnetic separation using Dynabeads ® GC Combo (Life Technologies). The separation of the parasite oocysts and the magnetic beads was carried out by changing the pH. The remaining 50 μl suspension was transferred to a glass slide, fixed with methanol and stained with FITC (fluoresceien isothiocyanate)-labelled monoclonal antibodies (BFT) and DAPI (4',6 -diamidino −2- phenylindole). Preparations were screened using a fluorescence microscope at appropriate wavelengths.

To identify *Cryptosporidium* species from the water samples, molecular investigations were performed in the United Kingdom’s *Cryptosporidium* Reference Unit at Public Health Wales. The reference unit received the only three remaining IMS-concentrated and purified aliquots (drawn 12 November 2013,) of river water (around 50 μl each) from the UBA, Germany. DNA was extracted and subjected to nested PCR SSU rRNA gene analysis as described previously [18].

## Results

### Epidemiological investigation

#### Initial assessment

In initial interviews with physicians in Halle 7 of 11 (64%) report an unusually high incidence of diarrhoea among their patients. Interviews with laboratories reveal that the different institutes do not have uniform eligibility criteria for cryptosporidiosis stool testing based; 3 out of 4 major laboratories serving Halle perform tests only upon specific request or automatically only in children <2 years (2/4 laboratories).

### Descriptive epidemiology

**Figure 2:**
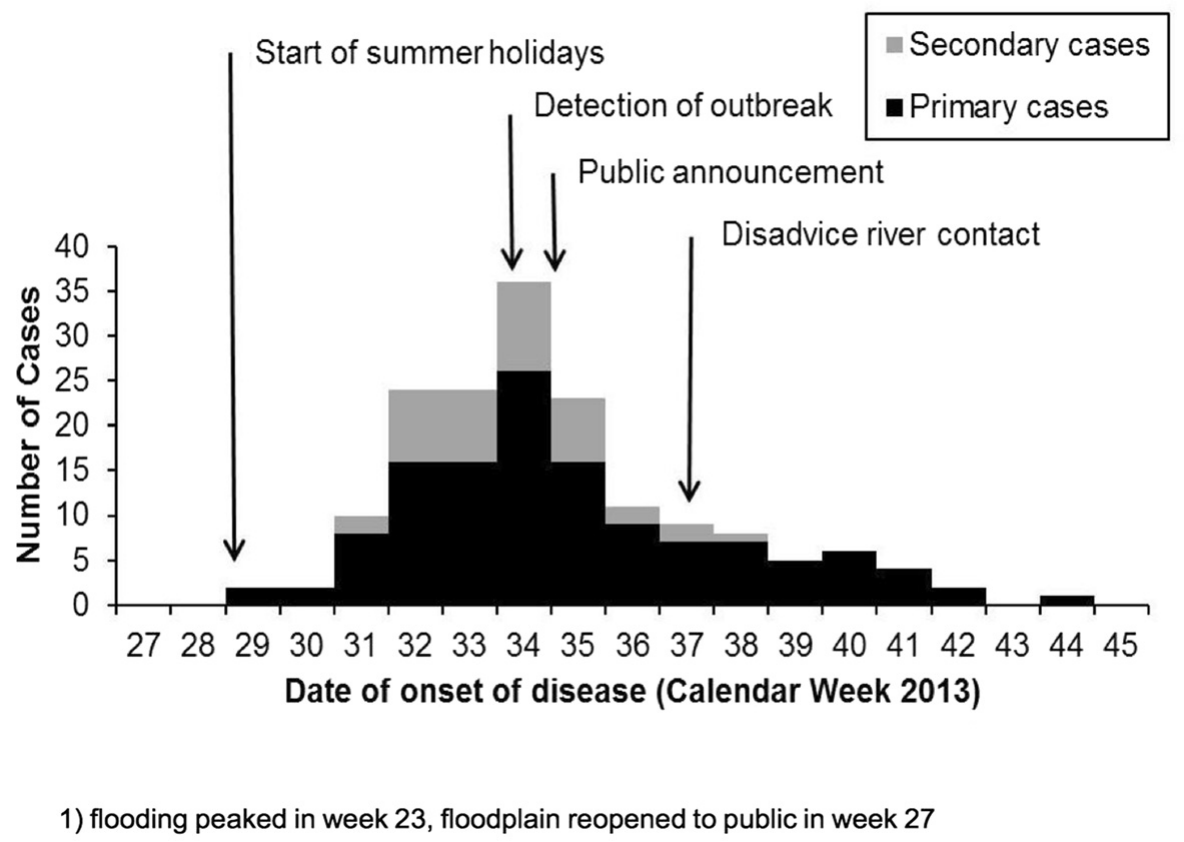
**Weekly distribution of date of onset of disease of reported Cryptosporidiosis cases during an outbreak in Halle (Salle) 2013 (n = 167).**

Altogether 167 cases were reported over a period of 17 weeks. First cases occurred during week 29 (Figure 2). Case numbers strongly increased from week 31, peaked in week 34 with 36 cases and strongly decreased afterwards. Cases appeared in all 5 districts of the city (Table 2). Incidence was highest in the central district and lowest in the eastern district. The central district comprises the most frequented recreational areas and the beach in the floodplain, while the eastern district is the only one which is not directly adjacent to the river. The cases’ median age was 8 years (range: 0–77 years), 81/167 (49%) patients were male, 60/167 (36%) were children attending kindergartens. Altogether 40/167 (24%) were classified as potential secondary cases, and 21/60 (35%) of the secondary cases were from kindergartens.

**Table 2:** **Distribution of cases in urban districts during the outbreak of Cryptosporidiosis in Halle (Saale), Germany 2013**

**District**	**Cases (% of total cases)**	**District population**	**Incidence (in 100,000)**
Centre	36 (21.6)	39,748	91
North	34 (20.4)	39,955	85
East	4 (2.4)	14,789	27
West	39 (23.4)	67,949	57
South	45 (26.5)	68,390	66
unknown	9 (5.4)		
Total	167 (100)	230,831	73

A cluster of 16 diarrhoeal cases in children confirmed as *Cryptosporidium* by laboratory analysis was detected in two kindergartens sharing the same premises. In the rural district Saalekreis which is surrounding the city of Halle, 23 cases were reported during weeks 27 through 47 compared to the annual average for 2008 to 2012: 5 cases/yr.

### Hypothesis generation

In exploratory interviews with 16 parents of primary cases, 12/16 (75%) reported activities (playing and picknicking) in the previously flooded areas, 10/16 (63%) reported using at least one of the three public swimming pools that tested positive for *Cryptosporidium* oocysts during the course of the investigation and 5/16 (31%) reported visits to either the public zoo or a different petting zoo. During the interviews it became clear that background rates of those activities were high due to the hot weather conditions and school holidays. Consumption of unboiled tap water, was reported by 9/16 (56%) while several respondents strictly denied this due to perceived bad taste. Based on these findings, an analytical epidemiological study of the exposure among diseased and non-diseased was launched to test the relevance of the following factors: activities in the previously overflowed floodplain, visits to public swimming pools and the zoo and consumption of tap water.

### Case control study

For the case control study we were able to contact 39 case patients of whom 20 (51%) met the study case definition and agreed to participate. Altogether 112 individuals responded as controls, 19 (17%) were excluded because of a history of diarrhoea after 1 July 2013, 15 (13%) because of a history of traveling outside Halle, 5 (4%) who did not provide a post code of their living address in Halle and another 12 (11%) who turned out to be actually not attending a kindergarten, resulting in 61 (54%) control persons. Cases and controls did not differ significantly in age and gender (p-value for gender: 0.2; p-value for age: 0.8).

Thirteen of the 20 (65%) cryptosporidiosis cases compared to 23/61 (38%) of the controls reported visits to previously flooded areas (Table 3) resulting in an odds ratio (OR) of 4.9 (95% CI 1.4-18.0) in univariable analysis. Nine cases (45%) indicated visits to the zoo (OR 2.6; 95% CI 0.9-7.6).

**Table 3:** **Exposures related to Cryptosporidiosis among infants during an outbreak in Halle, Germany 2013 (univariable and multivariable logistic regression)**

			Univariable*		Multivariable*	
**Exposure**	**Cases exposed (n/N, %)**	**Controls exposed (n/N, %)**	**OR**	**95% CI**	**OR**	**95% CI**
Stays in flooded area	13/20 (65)	23/61 (38)	4.92	1.35-18.02	5.50	1.40-21.56
Use of a swimming pool where *Cryptosporidium* oozysts were detected	5/20 (25)	17/61 (28)	0,8	0.26-2.62	-	-
Visits to the zoo	9/20 (45)	14/61 (23)	2.63	0.91-7.63	3.20	0.85-12.07
Consumption of tap water	9/20 (45)	41/61 (67)	0.40	0.14-1.22	-	-

*Adjusted for district of residence, OR: Odds ratio, 95% CI: 95%-Confidence interval.

Other variables were not related to cryptosporidiosis. In stratified analysis, 7/9 (78%) cases who visited the zoo, also visited the flooded areas. In multivariable analysis, visits to the floodplain remained the sole statistically significant risk factor (OR 5.5; 95% CI 1.4-22).

### Microbiological investigation

#### Human faecal samples

Of 170 specimens tested positive for *Cryptosporidium antigen* in primary diagnostics, 114 (67%) were sent to the NRC for species identification. In 106/114 (93%) presence of *Cryptosporidium* was detected by PCR, in 8/114 (7%) PCR was negative. Other coccidia such as *Cyclospora cayetanensis* or *Cystospora belli* as well as other diarrhoea-inducing protozoan parasites such as *Giardia lamblia* or *Entamoeba histolytica* were not detected. Molecular typing of 32/106 samples revealed that all of them represented *Cryptosporidium hominis* of the subtype IbA9G2 corresponding to GenBank accession number AY166807.

### Environmental samples

No *Cryptosporidium* oocysts were detected in the public water supply. Oocysts were found on 27 August 2013 in two public baths which were subsequently closed (Figure 2, Table 1). On 11 September a high concentration of 592 oocysts/100 litres was detected in an inner-city lateral channel of the river Saale called Mühlgraben, only a couple of metres from a frequently used river-beach and lawn for sunbathing and picnicking. In successive sampling during the following weeks, concentrations decreased but remained elevated (details in Table 1). Additionally, other sampling spots at the river Saale were confirmed positive (Table 1). Determination of *Cryptosporidium* species and subtype in the environmental samples (map identifiers 5, 8, 9) was not successful.

### Public health action

After confirmation of the outbreak, on 26 August 2013 the public health authorities informed the citizens of Halle via a press release about the outbreak and issued a precautionary boil-tap-water advisory. This measure was countermanded five days later, after considering the findings from the exposure interviews and the first tap water samples testing negative for oocysts. Two public swimming pools were positive and were closed. Furthermore, based on the results from the case control study and the water samples from the inner-city river sampling points, the health authorities informed the population about the risk associated with presence in the floodplain and advised against bathing and playing in or next to the river (13 September 2013).

The spillovers, where high concentrations of oocysts were found close by in the river water, were reassessed in October 2013 by the responsible municipal agency operating the system in Halle. No damages or dysfunctions were reported which would have allowed efflux of waste water in normal mode (none or only light rain) into the river.

## Discussion

We describe the largest recognized outbreak of cryptosporidiosis in Germany, which occurred in the city of Halle (Saale) and comprised 167 notified cases. The true size of the outbreak probably was much larger since many patients probably did not see a doctor and did not undergo laboratory testing for *Cryptosporidium*.

The outbreak began 6 weeks after the peak of an extensive river flooding which in the city inundated the whole floodplain as well as some adjacent streets and also affected and damaged the sewage systems. PCR confirmation was obtained for all but 8 (7%) of 114 samples recorded as positive for visual fluorescent antibody testing. The ability of PCR to determine the presence of *Cryptosporidium* in samples negative for visual analysis was not determined. We did not exlude these cases as the case definition was based on microscopy and antigen testing which are the most frequently used methods in primary diagnostics.

Epidemiological investigations provide evidence for exposure from human activities in the areas affected by the flood waters, including numerous playgrounds and the river beach, as a primary risk factor of infection of children – the most affected group in this outbreak. Repetitive water samples, taken two weeks to three months after the peak of the outbreak and in different locations, showed *Cryptosporidium* oocysts in the inner-city river, in some sites in very high concentrations. It appears plausible that children contracted infection when playing, bathing and also eating there. However, we did not ask what they did specifically in the floodplain.

However, interpretation of the case–control study is limited by the fact that exposure information was recorded differently among cases and controls (telephone and online) and it is not certain that the identified risk factors apply for other persons in the same way as for kindergarten children (study) group.

Based on preliminary results, local health authorities also released a notification of the risk in the floodplain (13 September) while the online questionnaire was still accessible (until 16 September), however only two controls and zero cases answered to the questionnaire or got interviewed after 13 September.

As *Cryptosporidium* oozysts were shown to being able to survive long periods in river water sediments*,* our findings cannot explain if the high concentrations of *Cryptosporidium* oocysts detected up to five months after the flooding, were exclusively caused by the preceding flooding or if ongoing sewage seepage or spills in a city with ongoing *Cryptosporidium* transmission contributed to this.

In any case, the oocyst counts measured at several spots (particularly no. 3–6 in the map) correspond to concentrations in open waters which according to WHO guidelines suggest pollution by wastewater [19]. In an analysis from a different river in German, Kistemann et al. detected median oocyst counts of only 4 oocysts/100 l (range 0–28) [20].

The involvement of wastewater in the aetiology of this outbreak is additionally supported by the nature of *C. hominis* which is almost entirely confined to humans [21-23].

However, evidence of the environmental findings is limited as species identification based on PCR-amplification in samples from the river water was not successful. Thus, source tracking to the river was limited to the findings of *Cryptosporidium* oocysts in the water. However, discrepancies between microscopic and molecular water sample investigations have been reported frequently [24].

To date, gastrointestinal disease outbreaks after river flooding in Central Europe usually have much smaller impact on public health than in countries with lower water and sanitation standards [25,26] and, waterborne outbreaks, even after flooding events were extremely rare in Germany [20,27]. The outbreak in Halle started about two weeks after public reopening of the previously closed floodplain. This could explain the outbreaks’ beginning two months after the flood. Furthermore, while most faecal pathogens may have died on the dried out floodplain during the period of very hot and dry weather in July 2013, *Cryptosporidium* oocysts have been described to survive several months particularly but not exclusively in moist environments [7,28]. The sodden and only slowly drying lawns of the floodplain parks likely provided such conditions. Infection has been described to occur after ingesting only 10 – 30 oocysts [29,30] and the concentrations detected in the river water close to the river beach and other locations in Halle were comparably high [4,31].

Decreasing case numbers coincide with the end of the summer holidays (23 August) and of the sunniest period which may underline the causative role of bathing in the river and/or ingestion via wet or muddy hands. It is questionable if the late public advice to restrain from bathing (13 September, week 37) based on our findings had a strong influence on decreasing case numbers as the bathing weather ended at this time. We need to consider that the participants were not asked what precisely they did in the floodplain (e.g. bathing, eating, playing) and our findings do not provide information on what the exact mechanism of transmission actually is.

Mason et al. report an outbreak caused by drinking water where <1 oocyst per 100 litres (size of water samples in our investigation) were detected in the drinking water [32]. Therefore, despite lack of evidence for tap water involvement in from initial patient interviews, we included this hypothesis in the case–control. However, the results provide no evidence for a causative role of tap water in this outbreak.

The Halle zoo was not overflowed by the flood. However, one of its exits leads directly to recreational areas in the floodplains, so that visits to the zoo and to the floodplain can be could easily combined. From the parasitological perspective, a causative role of the zoo would appear more likely if the relevant species here had not been *C. hominis*.

We did not find any evidence for food vehicles playing a role in this outbreak. Swimming pools have been frequently indicted as sources of infection during outbreaks of cryptosporidiosis [33-35]. Moreover, pool-to-pool transfer of oocysts by diarrhoeal swimmers has been described [36]. Whether the contaminated swimming pools in Halle were additional sources of infection remains unclear. In the case–control study among children, bathing in these pools was not associated with cryptosporidiosis. However, pools may have played a role for transmission to adults in this outbreak. Pool closures based on active sampling for oocysts and subsequent hygiene measures may have prevented additional gastroenteritis cases and shortened the outbreak.

## Conclusions

Even in countries with high standards of sanitation, river flooding can cause epidemics of gastroenteritis, when sewage systems overflow and pollute and water ways used by humans.

Severe flooding events have become more frequent in recent years and their frequency will probably further increase in Germany and other European countries due to climate change and stream straightening. Extreme water events such as flooding have been shown to be causative for the transmission of a number of waterborne diseases [37,38]. Also, the risk of gastrointestinal illness related to coastal and freshwater swimming was reported to be increased after heavy rainfall [39]. Evidence from this outbreak investigation suggests practical recommendations with regards to bathing within urban areas and preventive measures after river flooding: Particularly in urban areas, possible area and water contamination with persistent pathogens such as *Cryptosporidium* should also be considered and particularly open surface waters used for bathing should be tested also for parasites before re-opening to the public. River bathing sites should not be located near or closely downstream of potential sewage spillways, even if those are only operational on occasion. After flooding and during gastrointestinal outbreak situations of waterborne pathogens, sewage systems should be investigated for possible leakages.

In addition, the efforts to keep persons with diarrhoea or with little children who show such symptoms from using public swimming pools should be enhanced. In outbreak situations, swimming pools should be regularly evaluated for evidence of parasite oocysts.

Additionally, we found that physician ordering and laboratory routine testing schemes for *Cryptosporidium* are not very standardised. Thus validity of disease surveillance and outbreak detection based on laboratory detection is limited.

The molecular methods for detecting *Cryptosporidium* species and subtypes from environmental samples should be established in Germany. More research data on presence of *Cryptosporidium* oocysts in open surface water after heavy rains and in normal situtations is needed to better understand relevance for health threats after similar events.
